# Basketball expertise is associated with distinct relationship s among dynamic visual tracking, visuospatial working memory, and tactical decision-making

**DOI:** 10.3389/fpsyg.2026.1988470

**Published:** 2026-10-27

**Authors:** Jinyi Yu, Chang Xin, Yichao Diao

**Affiliations:** 1 Pai Chai University, Daejeon, Republic of Korea; 2 Jiuquan Vocational and Technical University, Jiuquan, Gansu, China; 3 Kyungil University, Gyeongsan, Republic of Korea; 4 Henan University, Kaifeng, Henan, China

**Keywords:** basketball, multiple-object tracking, perceptual-cognitive expertise, tactical decision-making, visual attention, visuospatial working memory

## Abstract

**Introduction:**

Tactical decision-making in basketball depends on the rapid extraction, maintenance, and use of changing spatial information, but the cognitive abilities associated with tactical performance may not be the same at different levels of expertise.

**Methods:**

In this cross-sectional study, 90 male basketball players were classified as expert, intermediate, or novice (*n* = 30 per group). Participants completed an MOT task, a computerized Corsi block-tapping task assessing VSWM, and a video-based TDM task. Group differences, within-group correlations, and expertise-moderated regression models were examined.

**Results:**

Experts showed higher MOT thresholds and TDM scores than both intermediate and novice players, and higher Corsi spans than novices. Within experts, MOT was positively associated with TDM (*r* = 0.535, FDR-adjusted *p* = 0.010), whereas within intermediate players, Corsi span was positively associated with TDM (*r* = 0.726, FDR-adjusted *p* < 0.001). The omnibus moderated-regression interaction block was significant, *F*(4, 80) = 3.331, *p* = 0.014. Direct pairwise slope contrasts showed that the MOT–TDM slope was stronger in experts than in intermediate players (Δ*B* = 7.210, Holm-adjusted *p* = 0.043) and that the Corsi–TDM slope was stronger in intermediate than in expert players (Δ*B* = 6.682, Holm-adjusted *p* = 0.042); the other pairwise slope contrasts did not survive multiplicity correction. Sensitivity analyses using Spearman correlations, HC3 robust standard errors, and influence diagnostics supported the main expert–intermediate contrasts.

**Discussion:**

These findings provide exploratory evidence that cognitive–decision associations differ across basketball expertise levels, particularly between expert and intermediate players. The cross-sectional design does not establish causal mechanisms or developmental transitions.

## 1 Introduction

Tactical decision-making (TDM) in basketball requires players to interpret rapidly changing spatial information under severe time constraints (Johnson, 2006; Raab et al., 2019; Guo and Wang, 2025). During offensive and defensive play, athletes must monitor the positions and movements of teammates and opponents, maintain an updated representation of the evolving game context, and select actions that fit the available affordances. Expert players generally outperform less-skilled individuals on sport-specific decision-making tasks, particularly when decisions are made from complex visual scenes. However, the cognitive basis of this advantage remains debated because superior tactical performance may arise from several interacting perceptual and cognitive processes rather than from a single general ability.

A central question in perceptual-cognitive expertise research is which cognitive processes are most closely linked to skilled tactical behavior (Scharfen and Memmert, 2019). Multiple-object tracking (MOT) is widely used as a laboratory measure of dynamic attentional allocation because it requires individuals to monitor several moving targets while ignoring distractors. In sport contexts, athletes often outperform non-athletes on MOT tasks, suggesting that sport participation may be associated with enhanced dynamic tracking ability (Gou and Li, 2023; Gou and Li, 2025a, b; Qiu et al., 2019; Styrkowiec et al., 2024). However, evidence for MOT differences within athletic populations remains mixed. Some studies report progressive improvements with increasing expertise (Silva et al., 2022), whereas others find weak or non-significant differences when perceptual familiarity, stimulus characteristics, or task constraints are controlled. It therefore remains unclear whether MOT reflects a stable capacity that scales with expertise or whether its relationship with sport performance depends on expertise level and decision context.

Tactical behavior also depends on the temporary maintenance and manipulation of spatial information (Furley and Memmert, 2012; Huffman et al., 2022; Ashford et al., 2021). Visuospatial working memory (VSWM) has therefore been proposed as another cognitive system that supports sport decision-making, particularly when athletes must integrate multiple spatial cues before selecting an action. Research in cognitive psychology and sport science suggests that higher working-memory capacity is often associated with better performance on complex decision-making tasks (Glavaš et al., 2023). In team sports, VSWM may be especially relevant when players must retain information about player locations, movement trajectories, and available passing or shooting options. Nevertheless, this relationship is unlikely to be uniform (Lex et al., 2015); the contribution of VSWM may vary with task demands, domain specificity, time pressure, and participant expertise.

These mixed findings raise a broader question about how perceptual tracking and working memory are functionally related during sport decision-making. A simple information-processing account would suggest that dynamic perceptual input is encoded through attentional tracking, maintained and manipulated in working memory, and then used to guide behavioral responses (Steiner et al., 2017). This sequence is considered here only as a theoretical possibility: the present study did not formally test mediation, temporal ordering, or a sequential MOT–VSWM–TDM pathway. Most previous work has emphasized mean-level group differences, whereas less is known about whether expertise is associated with different patterns of association among MOT, VSWM, and TDM.

Theoretical accounts of perceptual-cognitive expertise provide a basis for expecting such differences. Theories of chunking, long-term working memory, perceptual attunement and automaticity all suggest that extensive domain-specific practice changes how relevant information is encoded, organized, and used for action (Johnson, 2006; Raab et al., 2019; Zhu et al., 2024). Although these perspectives emphasize different mechanisms, they converge on the possibility that task-relevant perceptual information becomes more efficiently structured and more directly linked to action selection with increasing expertise. Neurocognitive findings showing reduced frontal executive engagement during expert domain-specific performance are consistent with this view, although they do not by themselves establish the behavioral structure of MOT–VSWM–TDM relationships.

Expertise-related differences in these associations need not imply a developmental reorganization of cognition. One possibility is that the relative associations of domain-general cognitive abilities with tactical performance vary across expertise levels because of differences in task demands, accumulated domain knowledge, perceptual familiarity, or selection effects. Cross-sectional comparisons cannot determine whether any observed pattern reflects training-related change, stable individual differences, reciprocal relationships, or other factors. Accordingly, expertise-related variation in MOT–VSWM–TDM associations was treated as an exploratory hypothesis rather than as an established stage-based mechanism.

The present study examined the relationships among MOT ability, VSWM capacity, and TDM performance in expert, intermediate, and novice basketball players. We tested whether the association structure among these variables differed across expertise levels without assuming a universal information-processing hierarchy. Three exploratory hypotheses guided the analysis: MOT was expected to show a comparatively strong association with TDM among experts; VSWM was expected to show a comparatively strong association with TDM among intermediate players; and statistically detectable associations were expected to be weaker among novices. These hypotheses concerned cross-sectional association patterns and did not constitute tests of developmental transition, cognitive reliance, mediation, or causal reorganization.

## 2 Materials and methods

### 2.1 Participants

Ninety male participants aged 18–25 years were assigned to expert, intermediate, or novice groups (*n* = 30 each) using a combination of competitive level and training history. Experts met a high-level competitive-status criterion: national first-level athlete certification, or membership in a university high-performance team together with extensive systematic training. Intermediate players were recruited from collegiate intramural teams or local amateur leagues and generally reported 3–7 years of structured training. Novices had limited basketball experience (no more than approximately 3 years) and did not meet the competitive criteria for the higher groups. Thus, training years and weekly training exposure were characteristics closely linked to the expertise classification rather than independent background predictors. All participants had normal or corrected-to-normal vision, were right-handed, and reported no neurological or psychiatric disorders or concussion within the previous 6 months. Right-handedness was used as a protocol-level standardization criterion; no laterality-specific hypothesis was tested. Participants were recruited between 1 July 2025 and 13 December 2025. Written informed consent was obtained before data collection. The study was approved by the Institutional Review Board of Jiuquan Vocational and Technical University (Approval No. 00015795) and conducted in accordance with the Declaration of Helsinki. An *a priori* power analysis in G*Power 3.1 indicated that 84 participants were required for a one-way ANOVA with three groups, assuming a conventional medium effect size of *f* = 0.25 (Cohen, 1988), *α* = 0.05, and 80% power. This calculation addressed the omnibus group comparison rather than the moderation analyses. To allow for potential data loss, 90 participants were recruited.

### 2.2 Apparatus and software

All tasks were administered individually in a quiet laboratory room with controlled illumination. Stimuli were displayed on a 24-inch LCD monitor (1920 × 1,080 pixels, 60 Hz refresh rate), with participants seated approximately 60 cm from the screen. Responses were collected using a standard keyboard and mouse. The experimental battery was programmed and presented using PsychoPy (version 2024.1.4) running on Windows 11, which provided millisecond-accurate stimulus presentation and response logging. A 10-point visual analog scale (1 = not at all fatigued, 10 = extremely fatigued) was administered verbally at the end of the session to monitor subjective fatigue.

### 2.3 Multiple-object tracking task

Dynamic tracking ability was assessed using a multiple-object tracking (MOT) task (Figure 1B). Participants tracked four target circles among four identical distractor circles (diameter = 0.8° visual angle) moving within a circular boundary. On each trial, all circles were stationary for 1,000 ms, after which the four targets turned blue for 1,000 ms and then changed back to red, and all circles moved for 5,000 ms. After movement stopped, participants identified the four targets by clicking them in any order. A three-down, one-up staircase procedure was used to estimate each participant’s tracking threshold, corresponding to approximately 79.4% accuracy. The initial speed was 4.0°/s, with a step size of 0.2°/s. Three consecutive correct responses increased speed, whereas one incorrect response decreased speed. The staircase terminated after 12 reversals, and the final threshold was calculated as the mean speed of the last eight reversals. Two practice blocks, each including six trials at 3.0°/s, preceded the formal task. The formal task included four blocks of 12 trials, for a total of 48 trials, separated by 30-s rests. The dependent variable was MOT speed threshold, with higher values indicating better dynamic tracking ability. Circle motion was generated online by the experimental program. Initial circle positions were sampled randomly under non-overlap and in-bounds constraints, and initial movement directions were sampled uniformly from 0 to 2π. Circles moved at a constant speed, with direction re-randomized approximately every 1 s. Circle–circle interactions followed an equal-mass two-dimensional elastic-collision rule: when the center-to-center distance became smaller than two radii, the velocity components along the collision axis were exchanged while tangential components were retained. When a circle reached the circular boundary, its position was returned to the point of contact and the velocity component normal to the boundary was reversed, producing specular reflection. Positions were updated using an 8-ms integration step, generating continuous trajectories composed of straight-line motion, random direction changes, boundary reflections, and inter-object collisions.

**Figure 1:**
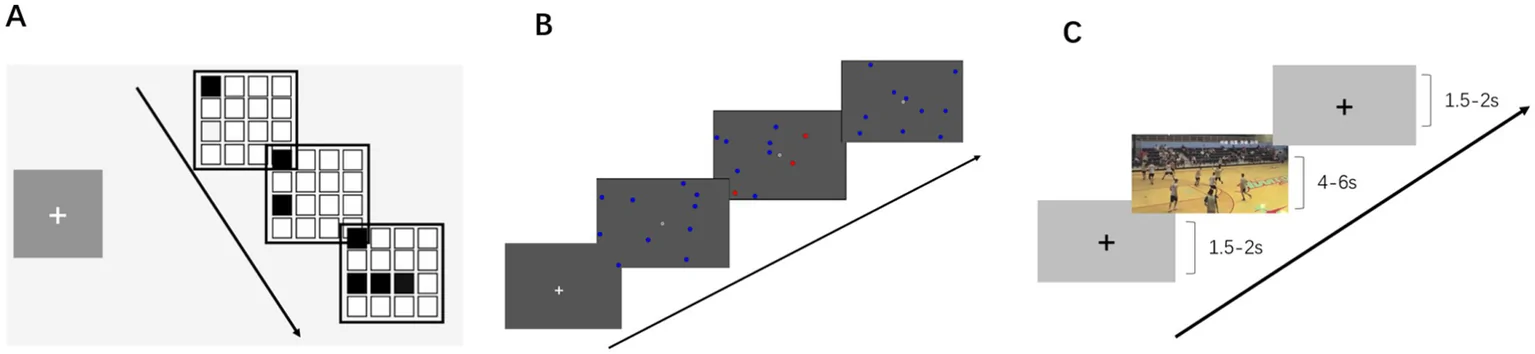
Overview of the experimental tasks: **(A)** computerized Corsi block-tapping task; **(B)** multiple-object tracking task; and **(C)** video-based tactical decision-making task.

### 2.4 Visuospatial working memory task

Visuospatial working memory (VSWM) capacity was assessed using a computerized Corsi block-tapping task (Figure 1A). Sixteen white squares were presented in an irregular spatial arrangement. On each trial, a sequence of squares changed from white to black one at a time for 500 ms each, after which participants reproduced the sequence by clicking the squares in the same order. The formal task used sequence lengths from 3 to 7 positions, corresponding to five difficulty levels. For each trial, target locations were sampled randomly without replacement from the 16 fixed spatial positions; therefore, no location was repeated within a sequence, and presentation order was randomized. A two-up, one-down staircase procedure was used to estimate memory span. The formal adaptive assessment began at the three-item level. Two consecutive correct responses increased the sequence length by one item, whereas one incorrect response decreased it by one item, within the programmed range of 3–7 items. The staircase terminated after six reversals, and the final Corsi span was calculated as the mean sequence length of the last five reversals, rounded to one decimal place. Averaging reversal levels provided a continuous threshold-like estimate that was less dependent on a single successful or failed sequence. Two practice trials with two-item sequences preceded the formal adaptive assessment. The dependent variable was Corsi span, with higher values indicating greater VSWM capacity.

### 2.5 Tactical decision-making task

Tactical decision-making (TDM) performance was assessed using a video-based simulation task (Figure 1C). Sixty video clips, each lasting 4–6 s, were selected from professional-league games and edited for standardized task presentation. The clips were presented from a wide-angle perspective that preserved the spatial configuration of the players relevant to the decision situation, and each clip ended with a freeze-frame at a critical decision point for the ball carrier. Clip extraction and selection were conducted in consultation with experienced basketball coaches and professional players (Reis et al., 2021) to ensure that the situations were representative of competitive basketball and appropriate for assessing tactical decision quality. The final set was equally distributed across five tactical contexts (Natsuhara et al., 2020), with 12 clips per context: fast break, pick-and-roll execution, penetration against help defense, ball reversal against set defense, and off-ball cuts. Because the task targeted decisions by the ball carrier, the stimulus set centered on offensive decision situations, with the defensive configuration represented by the positioning and movement of opponents within each clip.

After the freeze-frame, four response options appeared as text: Pass, Shoot, Drive, and a context-specific fourth option such as Hold/Reset or Pass back. Participants selected a response by keyboard press as quickly and accurately as possible. Three nationally certified basketball coaches independently evaluated each clip without the predefined response options, and only clips for which all three coaches agreed on the optimal action were retained. The study-specific graded rubric assigned 3 points to the unanimously identified optimal response, 2 points to a viable but less advantageous alternative that could still maintain or improve the tactical situation, 1 point to a contextually plausible but clearly lower-value alternative, and 0 points to an inappropriate choice for the displayed tactical configuration (Supplementary Table S1). Scores were clip-specific rather than globally attached to an action label; thus, Pass, Shoot, Drive, or the fourth option could receive different values in different clips depending on the tactical configuration. The total raw score (0–180) was linearly transformed to a 0–100 scale. Internal consistency was high (Cronbach’s *α* = 0.87). Six practice clips that did not overlap with the test clips preceded the formal task. Reaction time was recorded as a secondary procedural measure, but tactical decision quality, operationalized by the graded TDM score, was the prespecified primary outcome. Accordingly, reaction time was not included in the primary inferential analyses, which were designed to test the quality of tactical choices rather than speed–accuracy trade-offs.

### 2.6 Experimental procedure

Participants were tested individually in a single laboratory session lasting approximately 70–90 min. After providing informed consent, participants completed a demographic and training-history questionnaire and underwent a brief visual-acuity screening. The computerized tasks were administered in a fixed order with seated rest periods between tasks: 5-min rest/baseline preparation, MOT task, 5-min rest, Corsi task, 5-min rest, and TDM task. The fixed order followed the conceptual progression from dynamic perceptual tracking to visuospatial maintenance and finally to sport-specific tactical decision-making, but it was not counterbalanced. After completing the TDM task, participants reported subjective fatigue on the 10-point scale, were debriefed, and had the opportunity to ask questions. The fatigue rating was intended only as an end-of-session procedural check; because no pre-task fatigue score was obtained, it cannot quantify within-session change. Fatigue scores were not contained in the matched analysis dataset and were not used as covariates in the inferential analyses. No participant withdrew after beginning the protocol.

### 2.7 Statistical analysis

All analyses were conducted using IBM SPSS Statistics (version 27.0) and the PROCESS macro (version 4.2); revision-stage sensitivity analyses were independently reproduced from the matched participant-level dataset. A two-tailed *α* level of 0.05 was used. To make the inferential hierarchy explicit, the moderated regression testing expertise-related differences in the MOT–TDM and Corsi–TDM slopes was treated as the primary inferential analysis. Group mean comparisons were secondary analyses, whereas within-group correlations and Fisher r-to-z comparisons were used to characterize the association pattern.

Before the main analyses, data were screened for missing values and outlying observations. Normality was evaluated using skewness, kurtosis, and the Shapiro–Wilk test; homogeneity of variance was examined using Levene’s test. Multicollinearity in regression-based models was assessed using tolerance (>0.20) and variance inflation factor (VIF < 5). For the revision, scatterplots and regression influence diagnostics were additionally examined. Cook’s distance, externally studentized residuals, leverage, and DFBETAs were inspected for the moderated regression, and the two principal within-group correlations were checked using Cook’s distance and leave-one-out correlations. HC3 heteroskedasticity-robust standard errors and Spearman correlations were used as sensitivity analyses.

Group differences in demographic variables and primary outcome measures were examined using one-way ANOVAs. When homogeneity was violated, Welch’s F was consulted. Significant omnibus effects for the three primary outcomes were followed by Tukey HSD comparisons. To clarify the multiplicity procedure used in the submitted analysis, the nine Tukey pairwise *p*-values across MOT, Corsi, and TDM (three group comparisons per outcome) were treated as one family and adjusted using the Benjamini–Hochberg false discovery rate (FDR). Effect sizes for ANOVAs are reported as partial eta squared (partial *η*^2^).

Pearson correlation coefficients (*r*) with 95% confidence intervals were calculated separately within each expertise group for MOT threshold, Corsi span, and TDM score. The nine within-group correlations were treated as one exploratory family and adjusted using the Benjamini–Hochberg FDR. Spearman rank correlations were calculated as a sensitivity analysis. Between-group differences in correlation magnitude were examined with Fisher *r*-to-*z* tests; the nine Fisher comparisons (three association types × three group contrasts) were also FDR-adjusted as one exploratory family. Fisher tests were considered secondary to the moderated regression because they do not simultaneously adjust for age or the other cognitive predictor.

To test whether expertise moderated the relationships between cognitive measures and TDM, TDM score was regressed on standardized MOT threshold and Corsi span, expertise group, age, and the MOT × Group and Corsi × Group interactions. The novice group served as the reference category. The four-term interaction block was the primary test, followed by predictor-specific two-degree-of-freedom interaction blocks. Group-specific simple slopes were used to describe conditional associations but were not interpreted as evidence of between-group differences by themselves. To directly address between-group slope differences, planned expert–intermediate, expert–novice, and intermediate–novice linear contrasts were computed for each cognitive predictor from the fitted coefficient covariance matrix. Within each predictor, the three pairwise slope contrasts were Holm-adjusted. As robustness checks, HC3 standard errors were examined and the model was refitted after excluding observations with Cook’s D > 4/N.

The original *a priori* power analysis addressed the three-group ANOVA rather than moderation. A revision-stage sensitivity analysis was therefore used to quantify the implications of the achieved sample. With *n* = 30 per group, a two-sided correlation test at *α* = 0.05 had approximately 80% power only for |*r*| ≈ 0.48 or larger. For a comparison of two independent correlations with *n* = 30 per group, approximately 80% power requires a Fisher-z difference of about 0.76 (equivalent, for example, to comparing *r* ≈ 0.64 with *r* = 0). For the *N* = 90 moderated regression, approximately 80% power corresponded to an incremental four-term interaction effect of *f*^2^ ≈ 0.14. The study was therefore better powered for moderate than for small interaction or between-group association differences.

## 3 Results

### 3.1 Participant characteristics and descriptive statistics

Ninety male basketball players were included in the analysis, with 30 participants in each expertise group. Rechecking the matched analysis dataset reproduced the submitted inferential results and identified only minor rounding/transcription differences in the descriptive age and training-history values, which have been corrected in Table 1. The groups did not differ significantly in age, *F*(2, 87) = 0.226, *p* = 0.798. Training experience differed significantly, *F*(2, 87) = 299.981, *p* < 0.001, as expected because training history was closely related to the expertise classification.

**Table 1:** Participant characteristics and primary outcome measures by expertise group.

Variable	Expert	Intermediate	Novice
Age (years)	21.49 ± 1.64	21.19 ± 1.84	21.19 ± 2.07
Training experience (years)	10.36 ± 2.06	4.97 ± 1.01	1.64 ± 0.70
Weekly training (h)	11.68 ± 2.54	7.79 ± 1.74	3.27 ± 1.06
MOT threshold (°/s)	5.38 ± 0.67	4.82 ± 0.60	4.45 ± 0.65
Corsi span (items)	6.18 ± 0.89	5.61 ± 0.94	5.19 ± 0.86
TDM score (0–100)	77.60 ± 10.40	68.90 ± 9.80	60.20 ± 11.10

### 3.2 Group differences in primary outcome measures

One-way ANOVA showed a significant group effect for MOT threshold, *F*(2, 87) = 16.041, *p* < 0.001, partial *η*^2^ = 0.269. Tukey HSD comparisons, followed by BH-FDR adjustment across the nine pairwise comparisons for the three primary outcomes, indicated higher MOT thresholds in experts than in intermediate players (FDR-adjusted *p* = 0.007) and novices (FDR-adjusted *p* < 0.001); the intermediate–novice comparison was not significant (FDR-adjusted *p* = 0.080). Group means and 95% confidence intervals are shown in Figure 2A.

**Figure 2:**
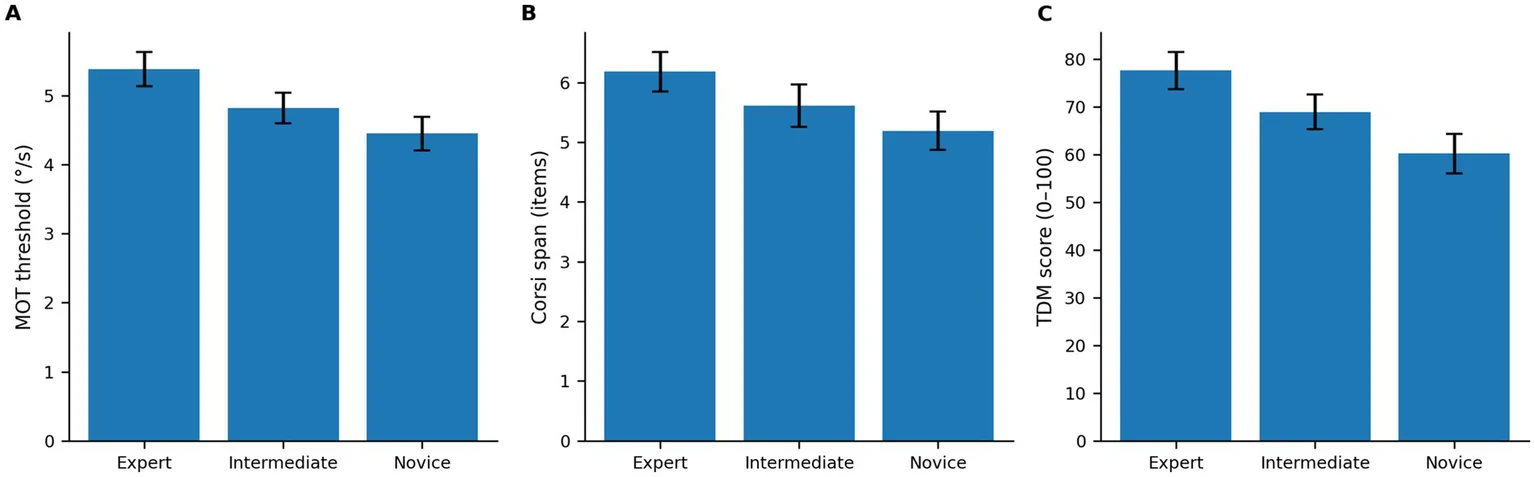
Primary outcomes across expertise groups **(A)** Multiple-object tracking (MOT) threshold **(B)** Corsi span and **(C)** Tactical decision-making (TDM) score. Bars show group means and error bars represent 95% confidence intervals calculated from the participant-level dataset.

Corsi span also differed across groups, *F*(2, 87) = 9.188, *p* < 0.001, partial *η*^2^ = 0.174. After the same multiplicity adjustment, experts had higher Corsi spans than novices (FDR-adjusted *p* < 0.001), whereas the expert–intermediate comparison did not reach the adjusted significance threshold (FDR-adjusted *p* = 0.054) and the intermediate–novice comparison was nonsignificant (FDR-adjusted *p* = 0.170; Figure 2B).

TDM scores differed significantly across groups, *F*(2, 87) = 20.805, *p* < 0.001, partial *η*^2^ = 0.324. Experts scored higher than intermediate players (FDR-adjusted *p* = 0.008) and novices (FDR-adjusted *p* < 0.001), and intermediate players scored higher than novices (FDR-adjusted *p* = 0.008; Figure 2C) (see Table 2).

**Table 2:** Group differences in primary outcome measures.

Variable	*F*	*p*	Partial *η*^2^
MOT threshold	16.04	< 0.001	0.269
Corsi span	9.19	< 0.001	0.174
TDM score	20.81	< 0.001	0.324

### 3.3 Within-group correlation analyses

Pearson correlations were examined separately within each expertise group (Figure 3). Table 3 reports r, 95% confidence intervals, raw *p*-values, and BH-FDR-adjusted *p*-values.

**Figure 3:**
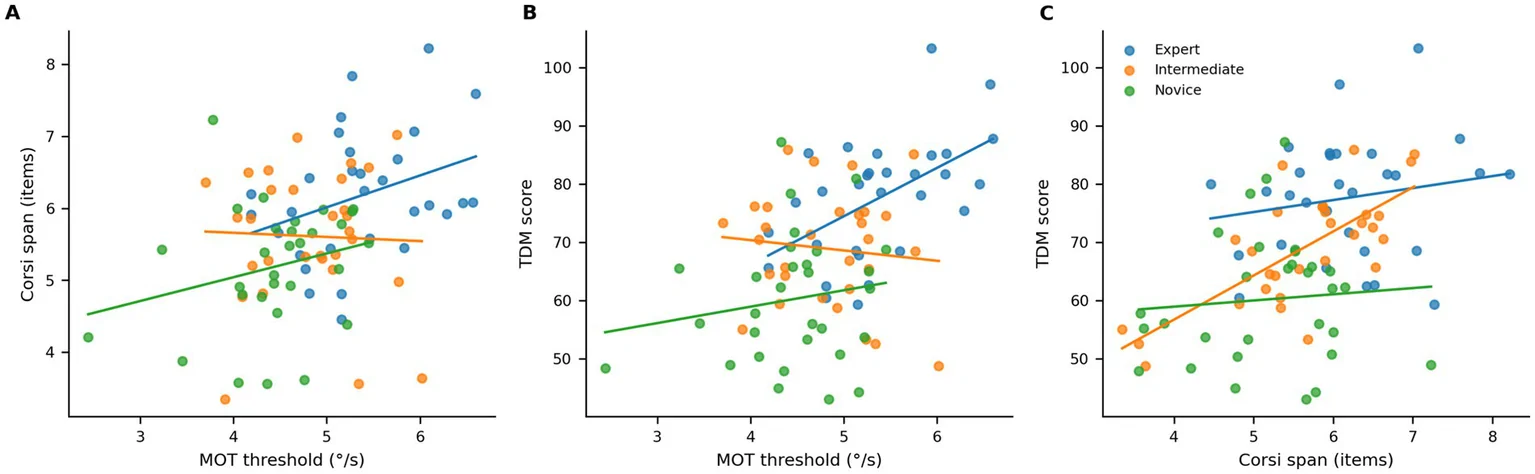
Within-group associations among multiple-object tracking (MOT), Corsi span, and tactical decision-making (TDM): **(A)** MOT threshold and Corsi span; **(B)** MOT threshold and TDM score; and **(C)** Corsi span and TDM score. Points are individual participants and lines are group-specific linear fits.

**Table 3:** Within-group Pearson correlations with 95% confidence intervals and Benjamini–Hochberg FDR-adjusted *p*-values.

Group	Association	*r*	95% CI	*p*	FDR-adjusted *p*
Expert	MOT–Corsi	0.332	[−0.032, 0.619]	0.073	0.218
Expert	MOT–TDM	0.535	[0.216, 0.750]	0.002	0.011
Expert	Corsi–TDM	0.176	[−0.197, 0.504]	0.353	0.576
Intermediate	MOT–Corsi	−0.037	[−0.392, 0.328]	0.847	0.847
Intermediate	MOT–TDM	−0.108	[−0.451, 0.262]	0.569	0.732
Intermediate	Corsi–TDM	0.726	[0.495, 0.861]	<0.001	<0.001
Novice	MOT–Corsi	0.249	[−0.122, 0.559]	0.184	0.414
Novice	MOT–TDM	0.165	[−0.208, 0.496]	0.384	0.576
Novice	Corsi–TDM	0.082	[−0.286, 0.430]	0.665	0.748

In experts, MOT threshold was positively associated with TDM score, *r* = 0.535, 95% CI [0.216, 0.750], *p* = 0.0023, FDR-adjusted *p* = 0.010. The MOT–Corsi association, *r* = 0.332, 95% CI [−0.032, 0.619], *p* = 0.073, and the Corsi–TDM association, *r* = 0.176, 95% CI [−0.197, 0.504], *p* = 0.353, were not statistically significant after FDR control.

In intermediate players, Corsi span was strongly associated with TDM score, *r* = 0.726, 95% CI [0.495, 0.861], *p* < 0.001, FDR-adjusted *p* < 0.001. MOT threshold was not significantly associated with Corsi span, *r* = −0.037, 95% CI [−0.392, 0.328], *p* = 0.847, or TDM score, *r* = −0.108, 95% CI [−0.451, 0.262], *p* = 0.569.

In novices, no statistically significant associations were detected: MOT–Corsi, *r* = 0.249, 95% CI [−0.122, 0.559], *p* = 0.184; MOT–TDM, *r* = 0.165, 95% CI [−0.208, 0.496], *p* = 0.384; and Corsi–TDM, *r* = 0.082, 95% CI [−0.286, 0.430], *p* = 0.665. The confidence intervals were wide, so these nonsignificant results should not be interpreted as evidence that the underlying associations are absent. Spearman sensitivity analyses reproduced the two principal associations (experts: MOT–TDM, *ρ* = 0.495, FDR-adjusted *p* = 0.024; intermediate players: Corsi–TDM, *ρ* = 0.652, FDR-adjusted *p* < 0.001).

### 3.4 Between-group comparisons of correlation coefficients

As a secondary analysis, Fisher r-to-z tests compared the group-specific correlations. After BH-FDR correction across all nine Fisher comparisons, the MOT–TDM association was stronger in experts than in intermediate players, *z* = 2.592, raw *p* = 0.0095, FDR-adjusted *p* = 0.029. The expert–novice and intermediate–novice MOT–TDM differences were not significant after correction (FDR-adjusted *p* = 0.256 and 0.401, respectively).

For Corsi–TDM, the correlation was stronger in intermediate than in expert players, *z* = 2.729, raw *p* = 0.0064, FDR-adjusted *p* = 0.029, and stronger in intermediate than in novice players, *z* = 3.078, raw *p* = 0.0021, FDR-adjusted *p* = 0.019. The expert–novice difference was not significant (FDR-adjusted *p* = 0.739). No MOT–Corsi between-group comparison was statistically significant after FDR correction. Because these Fisher tests are unadjusted for age and the other cognitive predictor, they were treated as secondary evidence.

### 3.5 Moderated regression analysis

The primary moderated regression accounted for 52.2% of the variance in TDM performance, *R*^2^ = 0.522, adjusted *R*^2^ = 0.469. Adding the four interaction terms significantly improved the model, *F*(4, 80) = 3.331, *p* = 0.0141, Δ*R*^2^ = 0.080. Predictor-specific block tests were also significant for MOT × Group, *F*(2, 80) = 3.413, *p* = 0.0378, and Corsi × Group, *F*(2, 80) = 3.669, *p* = 0.0299. With novices as the reference group, the MOT × Expert coefficient did not reach significance, *B* = 5.337, SE = 2.872, *p* = 0.0668, and MOT × Intermediate was nonsignificant, *B* = −1.873, SE = 2.937, *p* = 0.5254. Corsi × Intermediate was significant, *B* = 5.570, SE = 2.721, *p* = 0.0439, whereas Corsi × Expert was not, *B* = −1.112, SE = 2.806, *p* = 0.6930.

Direct pairwise slope contrasts were then computed from the fitted coefficient covariance matrix (Table 4). For MOT, the expert slope was steeper than the intermediate slope, Δ*B* = 7.210, 95% CI [1.476, 12.945], raw *p* = 0.0144, Holm-adjusted *p* = 0.043. The expert–novice and intermediate–novice MOT slope contrasts were not significant after Holm correction. For Corsi, the intermediate slope was steeper than the expert slope, Δ*B* = 6.682, 95% CI [1.390, 11.975], raw *p* = 0.0140, Holm-adjusted *p* = 0.042. The intermediate–novice contrast was nominally significant before correction (raw *p* = 0.0439) but did not survive Holm correction (adjusted *p* = 0.088), and the expert–novice contrast was nonsignificant. Thus, the clearest multiplicity-controlled between-group regression evidence concerned expert–intermediate differences, not a unique separation of either group from both other expertise levels.

**Table 4:** Group-specific simple slopes and direct pairwise slope contrasts from the moderated regression.

Analysis	Predictor	Group/contrast	B or ΔB	SE	*t*	*p*	Holm *p*	95% CI
Simple slope	MOT	Novice	0.985	2.050	0.481	0.6322	—	[−3.095, 5.065]
Simple slope	MOT	Intermediate	−0.888	2.091	−0.425	0.6722	—	[−5.049, 3.273]
Simple slope	MOT	Expert	6.322	1.985	3.184	0.0021	—	[2.371, 10.273]
Simple slope	Corsi	Novice	1.370	2.027	0.676	0.5012	—	[−2.665, 5.404]
Simple slope	Corsi	Intermediate	6.940	1.767	3.927	<0.001	—	[3.423, 10.457]
Simple slope	Corsi	Expert	0.258	1.973	0.131	0.8963	—	[−3.668, 4.183]
Pairwise contrast	MOT	Expert – Intermediate	7.210	2.882	2.502	0.0144	0.0432	[1.476, 12.945]
Pairwise contrast	MOT	Expert – Novice	5.337	2.872	1.858	0.0668	0.1336	[−0.378, 11.052]
Pairwise contrast	MOT	Intermediate – Novice	−1.873	2.937	−0.638	0.5254	0.5254	[−7.718, 3.971]
Pairwise contrast	Corsi	Expert – Intermediate	−6.682	2.659	−2.513	0.0140	0.0420	[−11.975, −1.390]
Pairwise contrast	Corsi	Expert – Novice	−1.112	2.806	−0.396	0.6930	0.6930	[−6.696, 4.472]
Pairwise contrast	Corsi	Intermediate – Novice	5.570	2.721	2.047	0.0439	0.0878	[0.156, 10.985]

Holm-adjusted *p*-values apply to the three direct pairwise slope contrasts within each predictor. Simple-slope *p*-values are conditional within-group estimates and are not tests of between-group differences.

Group-specific simple slopes were consistent with the bivariate pattern but were interpreted only as conditional within-group estimates. MOT was positively associated with TDM among experts, *B* = 6.322, SE = 1.985, 95% CI [2.371, 10.273], *p* = 0.0021, but not among intermediate or novice players. Corsi span was positively associated with TDM among intermediate players, *B* = 6.940, SE = 1.767, 95% CI [3.423, 10.457], *p* < 0.001, but not among experts or novices. Influence diagnostics identified five observations with Cook’s D > 4/N in the full moderated model, but no observation had an externally studentized residual with |r| > 3. Excluding the five Cook’s-D cases retained the overall interaction block, *F*(4, 75) = 3.306, *p* = 0.015. HC3 robust-standard-error analyses also retained the expert–intermediate slope differences for MOT (*p* = 0.003) and Corsi (*p* = 0.009). For the two principal within-group correlations, leave-one-out analyses retained statistical significance in every iteration (expert MOT–TDM *r* range = 0.472–0.584; intermediate Corsi–TDM *r* range = 0.676–0.771).

## 4 Discussion

This study examined whether the relationships among MOT, VSWM, and TDM vary across basketball expertise levels. Experts generally outperformed intermediate and novice players in MOT and TDM, and the within-group association pattern was heterogeneous: MOT was positively associated with TDM among experts, whereas Corsi span was strongly associated with TDM among intermediate players. However, the revised direct slope tests show that the strongest multiplicity-controlled between-group regression evidence concerns expert–intermediate differences. These results therefore support a cautious account of expertise-related heterogeneity in cognitive–decision associations rather than a categorical stage model or evidence of distinct cognitive mechanisms.

The superior MOT performance of experts is consistent with evidence that skilled athletes often show advantages in dynamic visual processing and attentional tracking (Mann et al., 2007; Voss et al., 2010; Faubert, 2013; Mangine et al., 2014; Ehmann et al., 2021; Jin et al., 2023; Liu et al., 2024; Alemanno et al., 2025). Importantly, evidence from athletes versus non-athletes should not be conflated with differences among expertise levels within athletic populations, because meta-analytic work suggests that domain-general cognitive advantages are typically smaller and less consistent than sport-specific perceptual-cognitive advantages (Voss et al., 2010; Kalén et al., 2021). In the present sample, experts exceeded both other groups in MOT, whereas intermediate and novice players did not differ significantly after multiplicity adjustment. A domain-general circle-tracking task may therefore capture some attentional demands relevant to basketball without mapping linearly onto competitive expertise. Basketball-specific tracking of players, ball movement, spacing, and tactical affordances may prove more sensitive to within-sport expertise differences (Roca et al., 2013; Gou et al., 2026).

For VSWM, experts showed higher Corsi spans than novices, whereas the expert–intermediate and intermediate–novice comparisons were not statistically significant after the prespecified multiplicity adjustment. The Corsi task measures maintenance of abstract spatial sequences rather than meaningful player configurations. It may therefore index a general cognitive resource relevant to some tactical situations without serving as a direct marker of basketball expertise. Studies of elite and sub-elite team-sport athletes likewise report mixed expertise differences in working memory and other executive functions, with effects varying by sport type, competitive level, and task demands (Verburgh et al., 2014; Huijgen et al., 2015; Krenn et al., 2018; Kalén et al., 2021; Haugan et al., 2025). By contrast, the video-based TDM task showed clear graded group separation, consistent with the greater domain specificity of tactical interpretation and accumulated sport knowledge (Mann et al., 2007; Roca et al., 2013; Gou et al., 2026).

Within experts, the MOT–TDM association remained significant after FDR control, was reproduced with Spearman correlation, and remained statistically significant in all leave-one-out analyses. The primary regression further showed that the MOT–TDM slope was significantly steeper in experts than in intermediate players after Holm correction. However, the expert–novice regression contrast was not significant. Accordingly, the data support an expert–intermediate difference in the MOT–TDM relationship, but they do not establish that experts uniquely differ from both other groups or that experts causally “rely” on MOT for tactical decisions. The observed association is compatible with prior evidence linking dynamic visual tracking to basketball-related performance and high-level processing of complex dynamic scenes (Faubert, 2013; Mangine et al., 2014), but longitudinal or experimental work is needed to evaluate mechanism.

Within intermediate players, Corsi span showed a strong association with TDM that was robust to FDR control, Spearman analysis, and leave-one-out checks. The direct multivariable slope contrast showed a stronger Corsi–TDM slope in intermediate than in expert players after Holm correction. Evidence for a difference between intermediate and novice players was less consistent: the secondary Fisher correlation comparison remained significant after FDR control, but the age-adjusted multivariable slope contrast did not survive Holm correction. The most defensible interpretation is therefore that VSWM–TDM coupling was especially pronounced in the intermediate group relative to experts, while its distinction from novices remains less certain. This pattern is broadly compatible with literature linking working-memory and executive processes to tactical behavior in team sports, while also emphasizing that such relationships vary across expertise levels and task demands (Furley and Memmert, 2012; Huijgen et al., 2015; Kalén et al., 2021; Haugan et al., 2025). It remains hypothesis-generating and does not demonstrate greater cognitive reliance on working memory at an intermediate developmental stage.

No statistically significant within-group associations were detected in novices, but the broad confidence intervals mean that this should not be interpreted as evidence of absence. General attentional or memory capacities may have weaker observable relationships with tactical choice when sport-specific representations are limited, because expert advantages are often most pronounced when tasks preserve domain-relevant perceptual information and contextual constraints (Mann et al., 2007; Roca et al., 2013). This explanation was not directly tested. Future studies should measure tactical knowledge alongside both domain-general and sport-specific cognitive tasks to distinguish these possibilities.

The significant omnibus interaction block indicates that the combined MOT–TDM and Corsi–TDM slope structure was not completely uniform across expertise groups. The direct pairwise regression contrasts refine that conclusion: after multiplicity correction, expert–intermediate differences were supported for both MOT and Corsi, whereas the other pairwise regression contrasts were not. The secondary Fisher analyses produced a somewhat broader pattern for Corsi–TDM, illustrating why within-group significance and unadjusted pairwise comparisons should not be treated as interchangeable with adjusted multivariable slope tests. The sensitivity analyses suggest that the overall interaction finding was not driven solely by a small number of influential observations, but the sample remains modest for interaction research.

The present findings do not test or establish a sequential pathway in which MOT supports VSWM and VSWM then supports TDM. No mediation or temporal-order model was fitted, and MOT and Corsi span were not significantly correlated within any group. Cross-sectional associations also cannot determine causal direction: tactical expertise could shape cognitive-task performance, cognitive capacities could contribute to tactical performance, both could influence one another, or both could reflect other training- and selection-related factors.

From an applied perspective, the observed patterns are best treated as hypothesis-generating signals for assessment design rather than as prescriptive training recommendations. Future studies should compare domain-general tasks with basketball-specific tracking and spatial-memory tasks within the same athletes, incorporate trial-level response time and speed–accuracy measures, and test whether the association patterns replicate prospectively. This emphasis on task representativeness and transfer is consistent with perceptual-cognitive training research showing that assessment and training effects are most interpretable when the informational constraints of the test resemble those of competitive performance (Pinder et al., 2011; Abernethy et al., 2012; Broadbent et al., 2015; Hadlow et al., 2018). The present data do not establish that training MOT or VSWM would improve tactical decision-making.

Several limitations should be acknowledged. First, the cross-sectional design precludes conclusions about developmental change, cognitive reorganization, or causal direction. Second, the original power analysis addressed the three-group ANOVA rather than moderation, and the sensitivity analysis indicates limited power for small correlations and interaction effects. Third, although FDR and Holm corrections were applied, multiplicity-related false-positive risk cannot be fully excluded. Fourth, MOT and Corsi are domain-general laboratory tasks and may not capture basketball-specific tracking or meaningful tactical configurations. Fifth, the TDM score used an expert-derived 3–2–1–0 rubric; although the optimal response was supported by unanimous expert agreement, the intermediate categories were not independently validated against an external criterion. Sixth, TDM was always administered last, so order or fatigue effects cannot be excluded, and the absence of a pre-task fatigue baseline prevents quantification of fatigue change. Seventh, all participants were young, male, and right-handed, limiting generalizability. Finally, despite supportive influence and robust-standard-error analyses, estimates remain imprecise with *n* = 30 per group and require independent replication.

## 5 Conclusion

The present study provides exploratory evidence that the relationships of dynamic visual tracking and visuospatial working memory with tactical decision-making vary across basketball expertise groups. MOT was positively associated with TDM within experts, whereas Corsi span was strongly associated with TDM within intermediate players. Direct multiplicity-controlled regression contrasts supported expert–intermediate differences for both relationships, while other pairwise slope contrasts were not consistently supported. These results should therefore be interpreted as selected expertise-related association differences rather than as evidence of distinct cognitive mechanisms, developmental transitions, or causal effects. Larger longitudinal studies using validated basketball-specific cognitive tasks are required to determine whether these patterns replicate and change with expertise development.

## Data Availability

The raw data supporting the conclusions of this article will be made available by the authors, without undue reservation.
