# Supplementary material for: Basketball expertise is associated with distinct relationship s among dynamic visual tracking, visuospatial working memory, and tactical decision-making
Source: Front Psychol. 2026 Oct 27;17:1988470. doi: 10.3389/fpsyg.2026.1988470 (PMC13747851; doi:10.3389/fpsyg.2026.1988470)
Supplement: Supplementary file 1 [file Table_1.docx]

**Supplementary Table S1. Operational interpretation of the study-specific TDM scoring categories.**

| **Score** | **Category** | **Operational interpretation** | **Evidence/limitation** |
| --- | --- | --- | --- |
| 3 | Optimal | The action unanimously identified by the three certified coaches as the best tactical choice for that specific clip. | Directly supported by unanimous coach consensus for the retained clip. |
| 2 | Suboptimal but effective | A viable alternative that could maintain or improve the tactical situation but was judged less advantageous than the optimal choice. | Expert-derived intermediate category; not independently validated against an external criterion. |
| 1 | Contextually acceptable but less advantageous | A tactically plausible option that did not exploit the primary advantage available in the displayed configuration. | Expert-derived intermediate category; not independently validated against an external criterion. |
| 0 | Inappropriate | A response judged inconsistent with the tactical configuration or clearly lower in tactical value than the available alternatives. | Study-specific expert-derived category. |

Note. Scores were clip-specific, not action-label-specific; the same response label could receive different scores in different tactical configurations.
